# HPV-DNA testing for patients with ASC-US helps identify the women who have a high risk for precancerous cervical lesions

**Published:** 2014

**Authors:** M Moarcăs, IC Georgescu, R Moarcăs, M Badea, M Cîrstoiu

**Affiliations:** * Obstetrics and Gynaecology Department, University Emergency Hospital, Bucharest; ** Obstetrics and Gynaecology Department, C.F. 2 Clinical Hospital, Bucharest; *** Micomi Clinic, Bucharest

**Keywords:** ASC-US, HPV testing, screening cervical lesions

## Abstract

**Background**. The cytological interpretation of ASC-US represents a category of morphologic uncertainty. For patients with this result, other tests are necessary in order to determine the risk for cervical lesions.

**Materials and methods**. 198 patients with ASC-US cytology have been analyzed between 2008 and 2013. All the patients included in the study have subsequently had a high oncogenic HPV testing and colposcopy risk. 103 (52%) patients tested positive for high risk HPV and 21 (10%) had associated colposcopy changes and precancerous and cancerous lesions identified through biopsy. 95 (48%) patients tested negative for HPV and none of these women had lesions at colposcopy.

**Results and discussion**. High oncogenic risk HPV testing was proven useful in identifying the patients with ASC-US cytology who are at high risk for cervical lesions (100% sensibility). In this study, the HPV testing had a negative predictive value of 100%, which uselessly renders a further colposcopy evaluation. HPV testing for women with ASC-US is not specific in identifying women with cervical lesions (Specificity 53%) and this results from a high prevalence of limited HPV infections in an age group which is less than 30 years old.

**Conclusions**: High risk HPV testing for women with ASC-US cervical cytology is useful in determining the risk for precancerous and cancerous cervical lesions. A positive result is associated with a high risk for cervical lesions (20%) and for these patients colposcopy is necessary. For women with a negative result, the risk for cervical lesions is practically null so colposcopy is not required.

**Abbreviations**: HPV = Human Papilloma Virus, ASC-US = Atypical Squamous Cells of Undetermined Significance, CIN = Cervical Intraepithelial Neoplasia, DNA = Deoxyribonucleic Acid, Pap = Papanicolaou test, LSIL = Low Grade Squamous Intraepithelial Lesion, HSIL = High Grade Intraepithelial Lesion.

## Background

Cervical cancer is the fourth most common cancer in women, and the seventh overall, with an estimated 528,000 new cases in 2012. In Romania the incidence is estimated at 4343 per 100,00 and the 5 year prevalence at 14834 per 100,000 (age-standardized rates per 100,000) [**1**]. Based on solid evidence, regular screening of appropriate women for cervical cancer with the Pap test reduces mortality from cervical cancer. The benefits of screening women younger than 21 years are small because of the low prevalence of lesions that will progress to invasive cancer. Screening is not beneficial in women older than 65 years if they have had a history of recent negative tests. Regular Pap screening decreases cervix cancer incidence and mortality with at least 80% [**2**-**4**]. In spite of these good results, cervical cytology has limitations: low reproducibility for ASC-US and LSIL, lack of specificity for precancerous lesions in ASC-US, highly variable results among laboratories, poor performance in detecting adenocarcinoma, frequent screening requirement (every 1 to 3 years) [**5**-**7**]. It is now known that cervical cancer is caused by sexually acquired infection with certain high-risk types of HPV, HPV16 and 18 being responsible for 70% of the cervical cancers and precancerous cervical lesions [**8**]. Recent studies have focused on the importance of high risk HPV testing in screening for cervical precancerous and cancerous cervical lesions. The need for other screening methods rose from the limitations associated with cervical cytology.

An ASC-US interpretation does not represent a specific cytological interpretation. Because of its morphologically equivocal nature, the inter- and intra-observer reproducibility of an ASC-US interpretation is less than that for the reliable, unequivocal cytological categories of LSIL and high-grade squamous intraepithelial lesion (HSIL) [**9**]. For patients with this result, other tests are necessary in order to determine the risk for precancerous cervical lesions. For women with positive high risk HPV test and ASC-US cytology, colposcopy is recommended because they have a 2-years risk of CIN3 and an invasive cervical cancer of 10% [**9**,**10**]. Studies have demonstrated that for women with ASC-US and negative high-risk HPV test, the risk for precancerous cervical lesions is very low, similar to women with negative cytology and HPV test. The absolute risk of CIN3 for women with ASC-US and negative HPV is of 0,28% at that moment and of 0,54% at 5 years [**11**,**12**]. Because this risk is less than 1% for these women, it is recommended to return to routine cervical screening [**9**].

## Materials and methods

198 patients with ASC-US cytology have been analyzed in Micomi Clinic, between 2008 and 2013. All the patients included in the study have subsequently had a high oncogenic risk HPV testing and colposcopy. 103 (52%) patients tested positive for high risk HPV and 21 (10% of all women) had associated colposcopy changes and precancerous and cancerous lesions were identified on biopsy. 95 (48%) patients tested negative for high risk HPV and none of these women had identifiable lesions at colposcopy (**Table 1**).

**Table 1:** Study population quantification according to HPV and Cervical lesion status

		Cervical Lesion		
		Present	Absent	Total
High Risk HPV Test	Positive	21	82	103
	Negative	0	95	95
	Total	21	177	198

The study population comprised women with ages between 21 and 60 years old, with a mean age of 37. **Graph 1** shows the population distribution according to age and high-risk HPV status. We do not have information regarding the type of HPV infection, whether it was persistent or not. The women who were identified with cervical lesions were between 29 and 55 years old, with a mean age of 38.

**Fig. 1:**
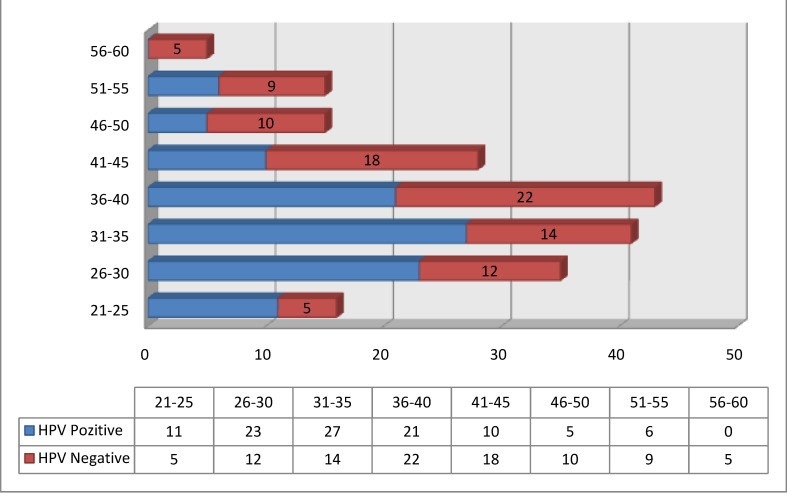
The study population distribution according to the age group and HPV status

## Results and discussion

High oncogenic risk HPV testing risk has proven useful in identifying the patients with ASC-US cytology who are at high risk for cervical lesions (100% sensibility). In this study, HPV testing had a negative predictive value of 100%, which would uselessly render a further colposcopy evaluation. HPV testing for women with ASC-US is not specific in identifying women with cervical lesions (Specificity 53%) and these results from a high prevalence of limited HPV infections in the age group of less than 30 years old. HPV infections usually clear up without any intervention within a few months after acquisition, and about 90% clear within two years [**8**]. Taking into consideration the fact that HPV investigation is a screening test it is preferable to have a high sensitivity with a lower specificity than a low sensitivity. 

The statistical significance of these findings is limited by the small number of women included in the study. Also clinicians should exercise caution when extending these findings to the general population as the sample analyzed in this study has a higher economic status being formed by women who attended private medical care. 

## Conclusions

High risk HPV testing for women with ASC-US cervical cytology is useful in determining the risk for precancerous and cancerous cervical lesions. A positive result is associated with a high risk for cervical lesions (20%) and for these patients colposcopy is necessary. For women with a negative result the risk for cervical lesions is practically null so colposcopy is not required and these women can return to routine screening. HPV testing for women with ASC-US reduces the number of colposcopies that require highly trained physicians and can be uncomfortable for women.
